# Effective care for mothers and their babies during humanitarian crises

**DOI:** 10.2471/BLT.25.295391

**Published:** 2026-01-01

**Authors:** Olive Cocoman, Hannah Tappis, Elaine Scudder, Harriet Ruysen, James McQuen Patterson, Shirley Mark Prabhu, Tomomi Kitamura, Mollie Fair, Muna Abdullah, Khalid Siddeeg, Mohammed Afifi, Janet Kayita, Sophie Chimwenje Khumbizeni, Valérie Marcella Zombre Sanon, Khadidja Amadaye Abgrene, Rima Chaya, Tala Rammal, Majid El Nour, Kathleen Mitchell, Catrin Schulte-Hillen, Gagan Gupta, Allisyn Moran

**Affiliations:** a Department of Maternal, Newborn, Child and Adolescent Health and Ageing, World Health Organization, Geneva, Switzerland.; b Jhpiego - an affiliate of Johns Hopkins University, Baltimore, United States of America (USA).; c International Rescue Committee, New York, USA.; d Department of Infectious Disease Epidemiology and International Health, Faculty of Epidemiology and Population Health, London School of Hygiene & Tropical Medicine, Keppel Street, London WC1E 7HT, England.; e Health Section, United Nations Children’s Fund, New York, USA.; f Health and HIV Section, United Nations Children’s Fund, Middle East and North Africa Regional Office, Amman, Jordan.; g Health and HIV Section, United Nations Children’s Fund, West and Central Africa Regional Office, Dakar, Senegal.; h Sexual and Reproductive Health Programme, United Nations Population Fund Arab States Regional Office, Cairo, Egypt.; i Sexual and Reproductive Health Programme, United Nations Population Fund East and Southern Africa Regional Office, Johannesburg, South Africa.; j Department of Healthier Populations, World Health Organization Regional Office for the Eastern Mediterranean, Cairo, Egypt.; k Family and Reproductive Health, World Health Organization Regional Office for Africa, Brazzaville, Republic of Congo.; l Reproductive Health Department, Ministry of Health, Lilongwe, Malawi.; m Family Health Division, Ministry of Health and Public Hygiene, Ouagadougou, Burkina Faso.; n Reproductive Health Division, Ministry of Public Health, N’Djamena, Chad.; o Health Section, United Nations Children’s Fund, Beirut, Lebanon.; p Sexual and Reproductive Health Programme, United Nations Population Fund, Khartoum, Sudan.; q Sexual and Reproductive Health in Emergencies, United Nations Population Fund, New York, USA.

Although global maternal and neonatal mortality have declined over recent decades, progress has stalled in contexts affected by conflict, fragility and displacement.^1^^–^^3^ The 29 countries with United Nations humanitarian response plans or flash appeals at the start of 2025 accounted for slightly less than one third of global births but contributed an estimated 58% of maternal deaths, 41% of stillbirths and 39% of newborn deaths.^2^^–^^4^ These figures likely underestimate the burden in acute crises, where data are usually scarce.^5^^,^^6^

The *Global humanitarian overview*^7^ was revised in June 2025 to acknowledge declines in foreign aid, increasing attacks on health and humanitarian workers, and deepening instability. Despite these challenges, a 2022 United Nations country-level survey demonstrated that even in crisis settings, progress is possible when maternal and newborn health are prioritized, financed and integrated into national systems and preparedness plans.^8^ We suggest that integration also requires coordinated action, accountability and sustained investment aligned with three principles.

First, strengthening leadership and prioritizing a continuum of care approach. Maternal and newborn health services in crisis settings face fragmented implementation and chronic underfunding. While some countries meet antenatal care coverage targets, postnatal care remains underprioritized, and stillbirth prevention is often absent from monitoring and planning frameworks.^8^ Meaningful progress requires a continuum of care approach, from pre-conception care to the postnatal period and beyond. Data are also essential to make maternal and newborn health visible. 

Humanitarian and development actors must coordinate technical support aligned with national priorities. Initiatives such as *Every woman every newborn everywhere* provide technical assistance to over 55 countries to strengthen subnational planning and implementation.^9^ These efforts require predictable, multiyear financing, pooled funds or public–private partnerships to ensure continuity of essential maternal and newborn health services. 

Second, integrating emergency response and recovery. Humanitarian emergencies require phased response strategies balancing immediate lifesaving interventions with longer-term system recovery. During acute emergencies, the minimum initial service package for sexual and reproductive health in crisis settings ensures essential standards for skilled birth attendance, emergency obstetric and newborn care, prevention of unintended pregnancies and care for small and sick newborns.^10^ Availability of essential commodities, including interagency reproductive health kits, is critical. As crises stabilize, expanding access to integrated primary care, including quality antenatal and postnatal care as well as family planning, can mitigate health risks and revive community trust that is essential for health system recovery.

However, evaluations show persistent gaps: limited access to safe abortion care aligned with national legislation, insufficient emergency obstetric and newborn care, incomplete referral systems, supply-chain disruptions and inadequate funding.^11^ While national-level commitment to the minimum initial service package has grown, subnational implementation lags.^11^ Stronger partnerships are needed among humanitarian and development actors, local governments and host communities to reach the most vulnerable. Emergency interventions should strengthen, not bypass, national systems.

Third, protecting and sustaining the health workforce. In crisis settings, health workers face insecurity, constrained resources and personal risk, making their protection and retention both a humanitarian duty and a prerequisite for system recovery.

International humanitarian law requires protection of health workers and facilities. Governments, humanitarian agencies and parties to the conflict must ensure safe working conditions, adequate remuneration and psychosocial support, as well as investments in supervision, training and supply chains. Surge and rotational deployments, task-shifting and peer-support networks can strengthen workforce capacity in insecure environments.

Resolution WHA77.5,^1^ adopted in 2024, calls for accelerating progress towards reducing maternal, newborn and child mortality. The resolution was endorsed by 194 countries,^1^ reflecting collective resolve amid profound global upheaval and recognition that achieving global health targets depends on protecting every woman and newborn whether in conflict zones, displacement camps or communities affected by extreme weather events. Enacting this resolution is a humanitarian imperative and a prerequisite for global health equity. To do so, governments should convert commitments into actionable, budgeted national and subnational plans supported by sustained financing and robust monitoring, while donors and partners should align humanitarian and development funding to provide evidence-based guidance, operational support and accountability mechanisms.
